# Supplementary figures and images for: Curcumin plays a synergistic role in combination with HSV-TK/GCV in inhibiting growth of murine B16 melanoma cells and melanoma xenografts
Source: PeerJ. 2019 Sep 20;7:e7760. doi: 10.7717/peerj.7760 (PMC6756137; doi:10.7717/peerj.7760)

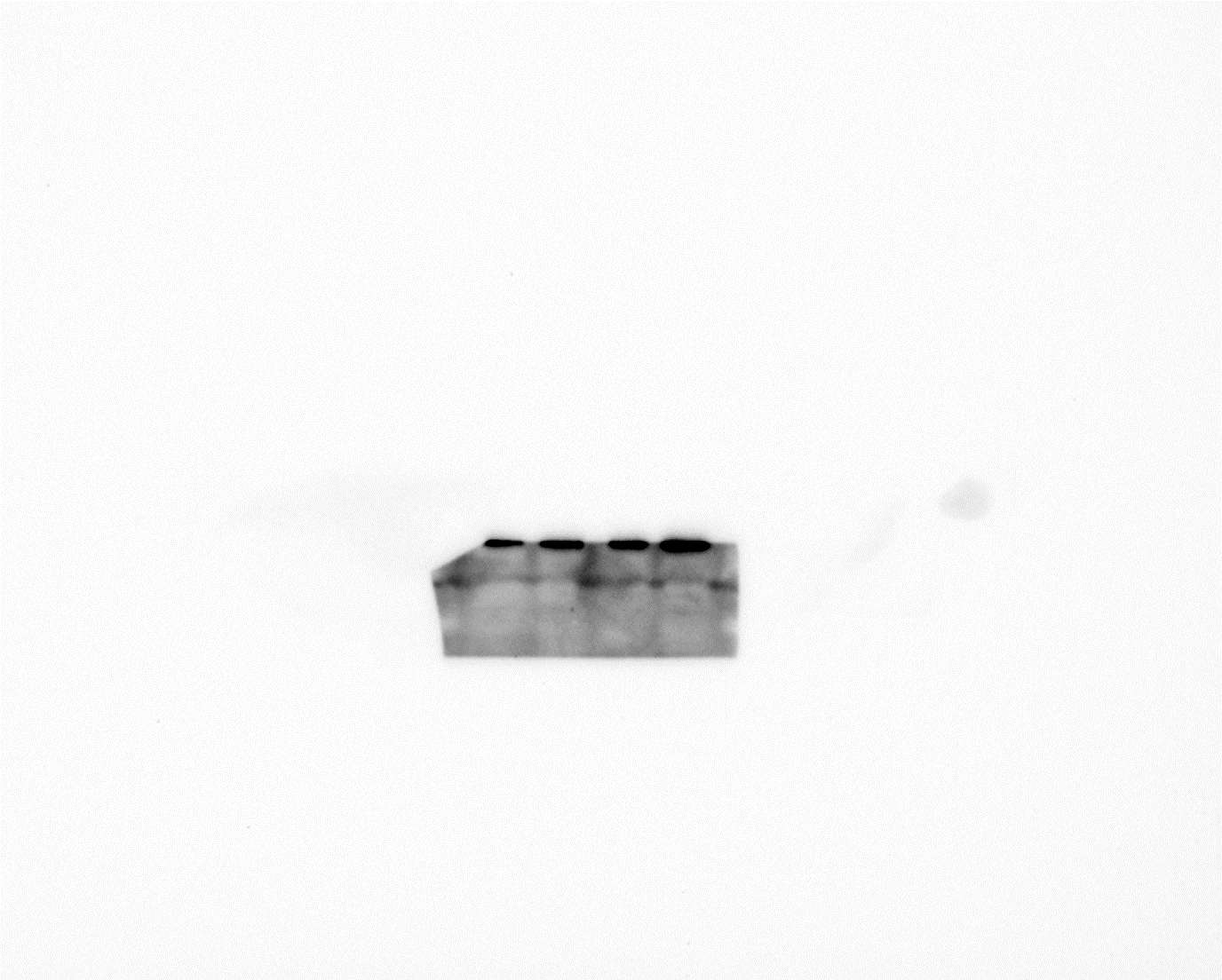

Supplement: Data S1 — The raw data of the MTT assays, the flow cytometry assays, Western Blot assays, and the animal studies. [file peerj-07-7760-s001.zip › raw data/Western Blot/Cx32/Cx32-1.tif]

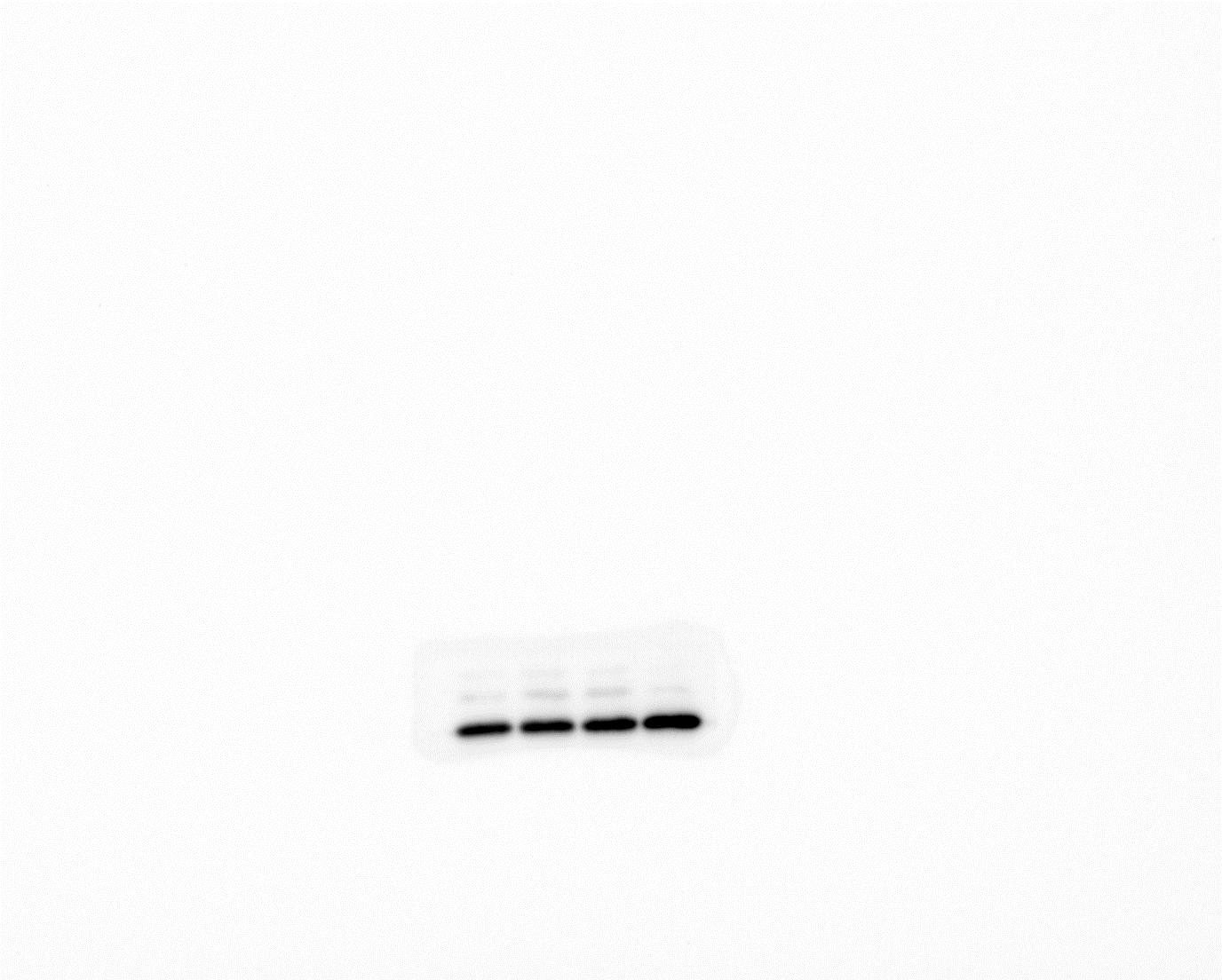

Supplement: Data S1 — The raw data of the MTT assays, the flow cytometry assays, Western Blot assays, and the animal studies. [file peerj-07-7760-s001.zip › raw data/Western Blot/Cx32/Cx32-2.tif]

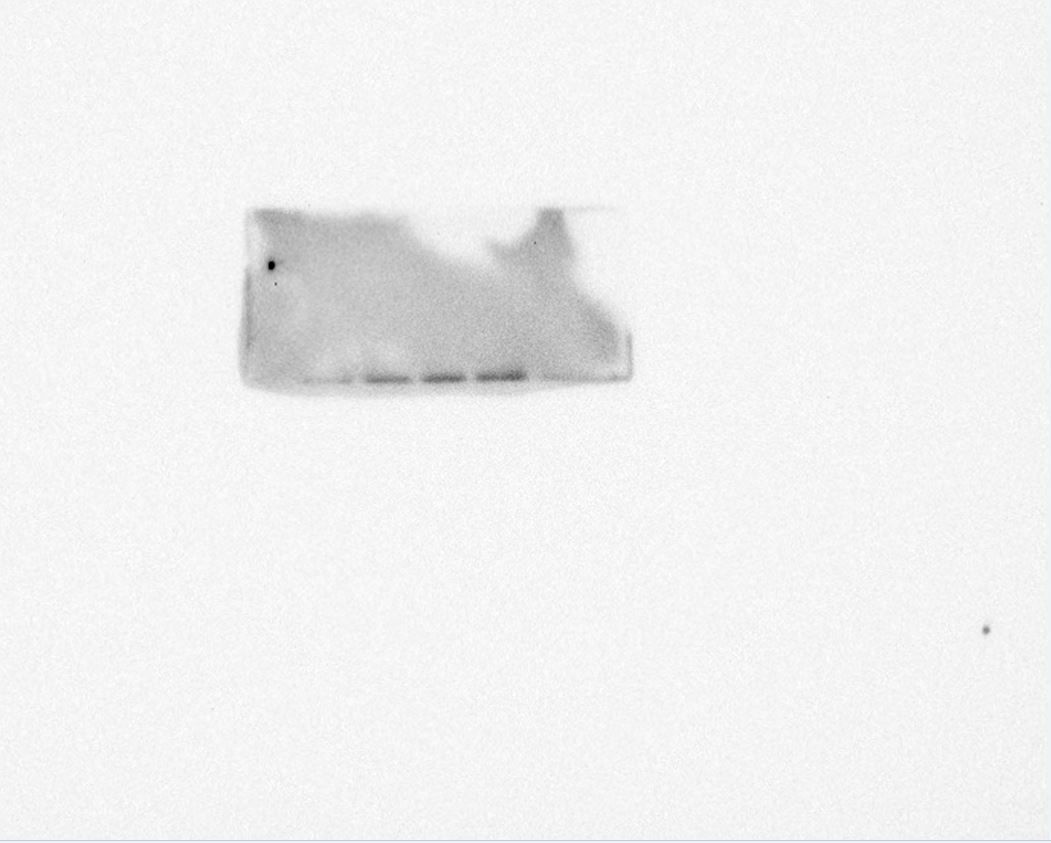

Supplement: Data S1 — The raw data of the MTT assays, the flow cytometry assays, Western Blot assays, and the animal studies. [file peerj-07-7760-s001.zip › raw data/Western Blot/Cx32/Cx32-3.JPG]

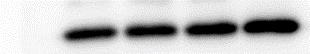

Supplement: Data S1 — The raw data of the MTT assays, the flow cytometry assays, Western Blot assays, and the animal studies. [file peerj-07-7760-s001.zip › raw data/Western Blot/Cx32 2.jpg]

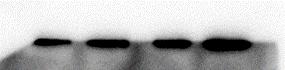

Supplement: Data S1 — The raw data of the MTT assays, the flow cytometry assays, Western Blot assays, and the animal studies. [file peerj-07-7760-s001.zip › raw data/Western Blot/Cx32-1.jpg]

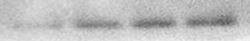

Supplement: Data S1 — The raw data of the MTT assays, the flow cytometry assays, Western Blot assays, and the animal studies. [file peerj-07-7760-s001.zip › raw data/Western Blot/Cx32-3.JPG]

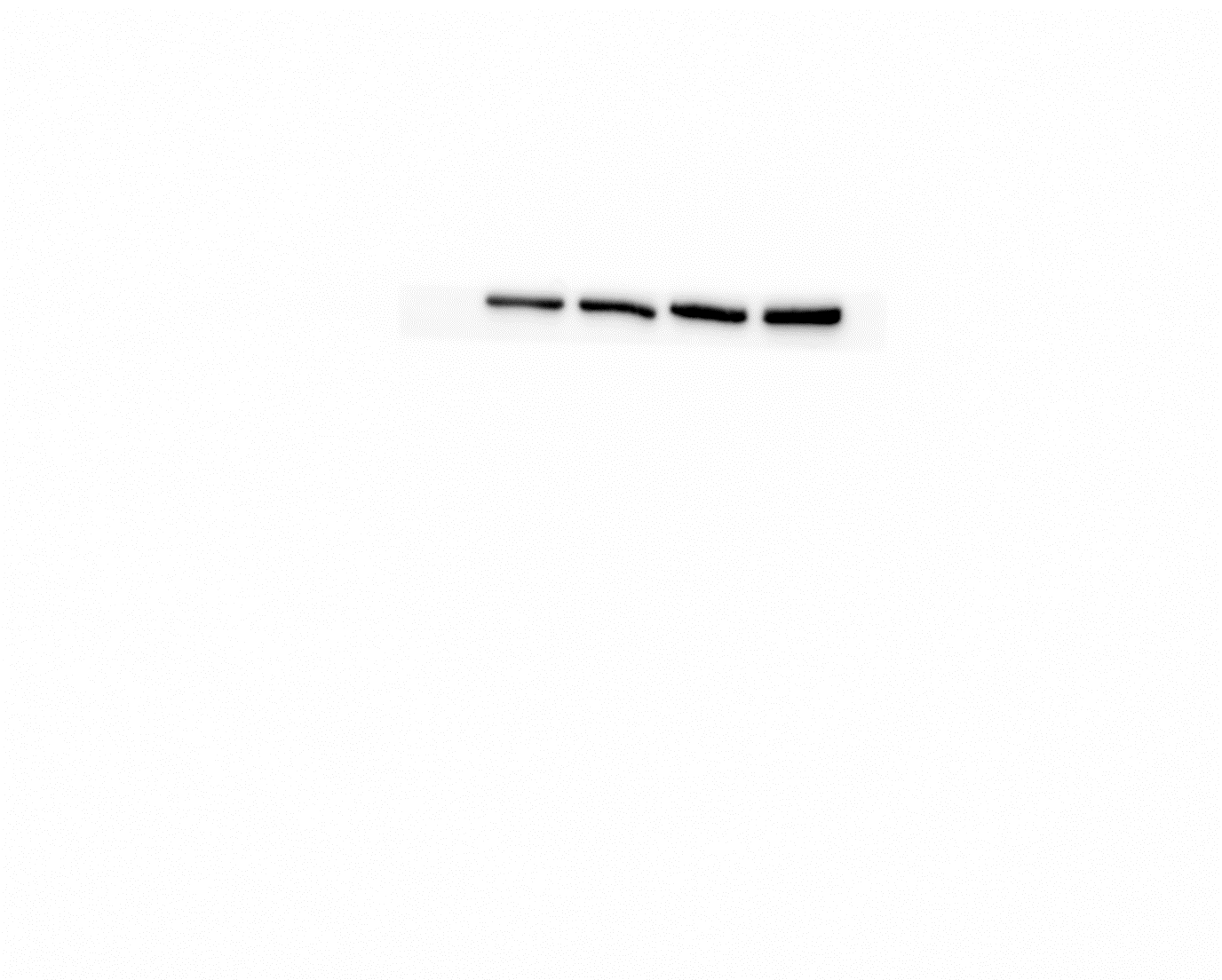

Supplement: Data S1 — The raw data of the MTT assays, the flow cytometry assays, Western Blot assays, and the animal studies. [file peerj-07-7760-s001.zip › raw data/Western Blot/Cx43/Cx43-1.tif]

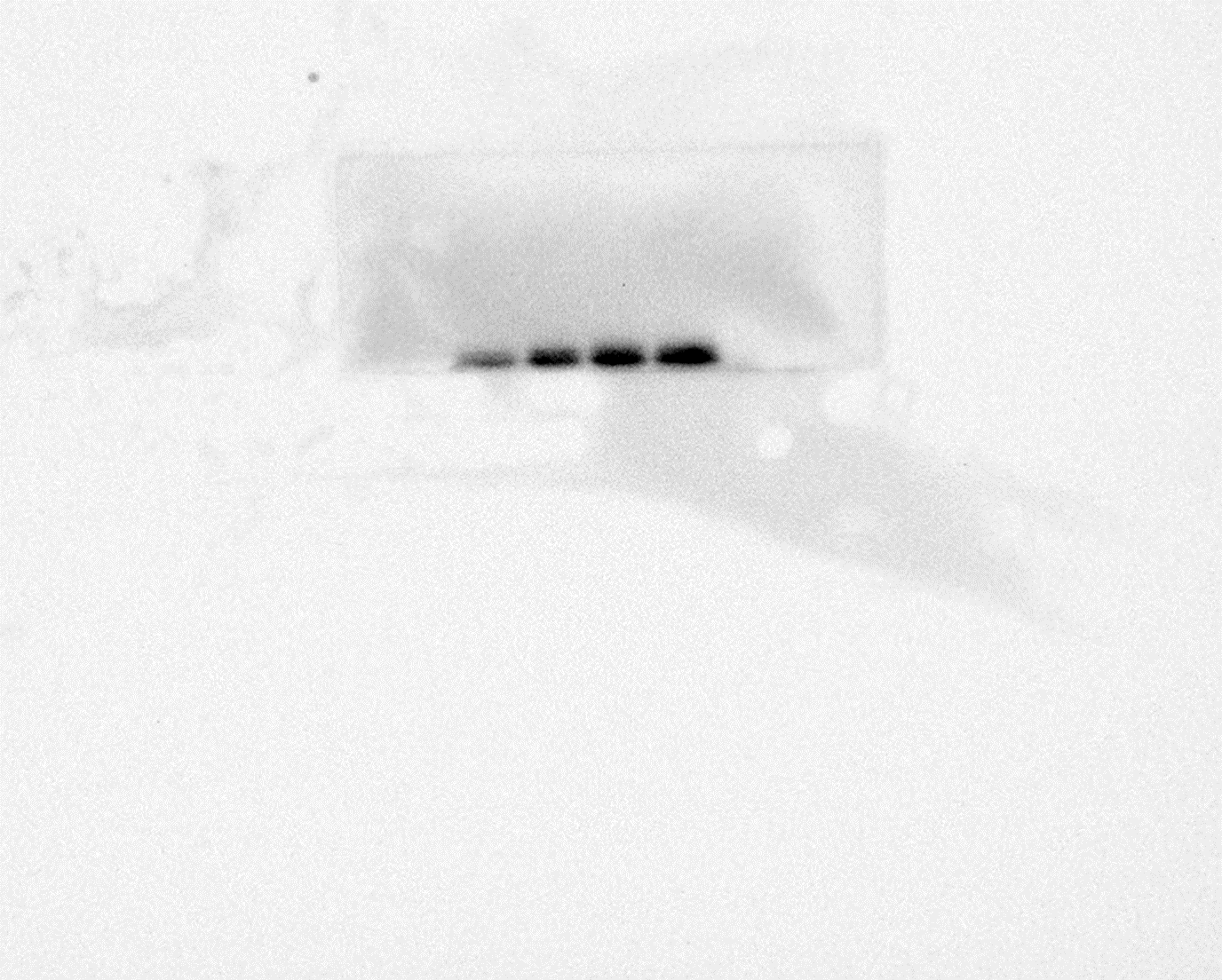

Supplement: Data S1 — The raw data of the MTT assays, the flow cytometry assays, Western Blot assays, and the animal studies. [file peerj-07-7760-s001.zip › raw data/Western Blot/Cx43/Cx43-2.tif]

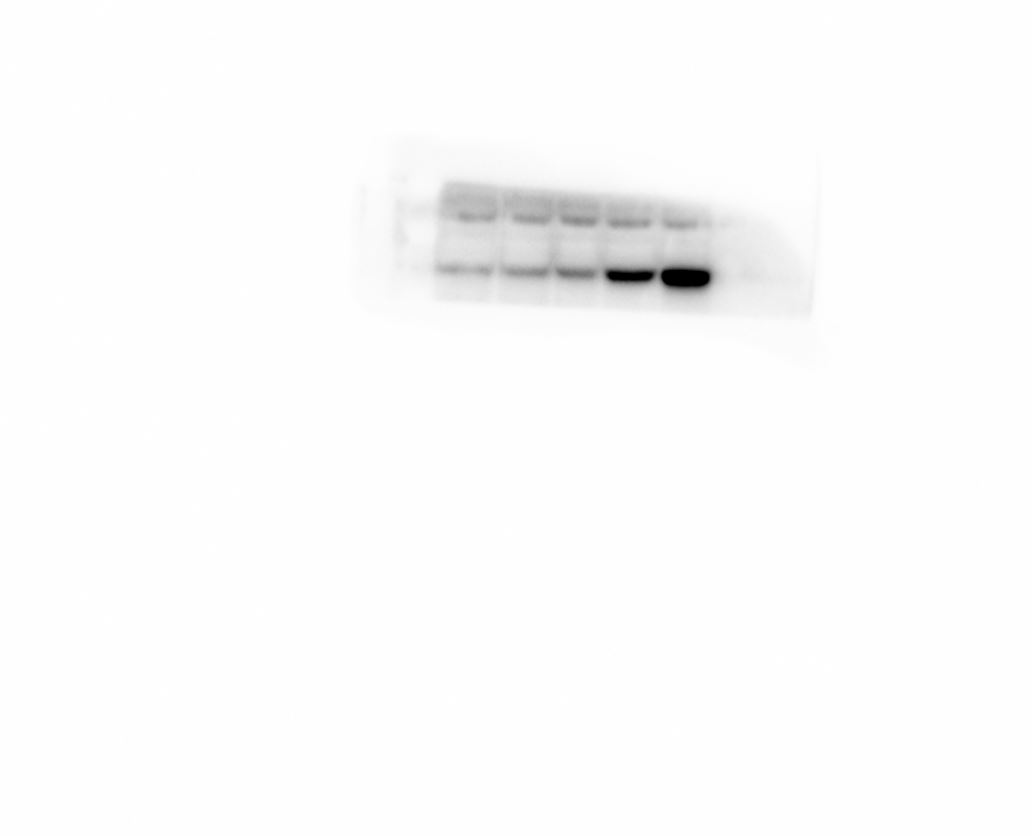

Supplement: Data S1 — The raw data of the MTT assays, the flow cytometry assays, Western Blot assays, and the animal studies. [file peerj-07-7760-s001.zip › raw data/Western Blot/Cx43/Cx43-3.JPG]

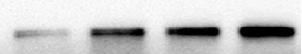

Supplement: Data S1 — The raw data of the MTT assays, the flow cytometry assays, Western Blot assays, and the animal studies. [file peerj-07-7760-s001.zip › raw data/Western Blot/Cx43 1.jpg]

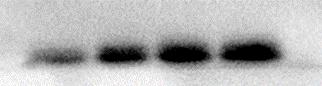

Supplement: Data S1 — The raw data of the MTT assays, the flow cytometry assays, Western Blot assays, and the animal studies. [file peerj-07-7760-s001.zip › raw data/Western Blot/Cx43 2.jpg]

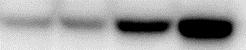

Supplement: Data S1 — The raw data of the MTT assays, the flow cytometry assays, Western Blot assays, and the animal studies. [file peerj-07-7760-s001.zip › raw data/Western Blot/Cx43 3.jpg]

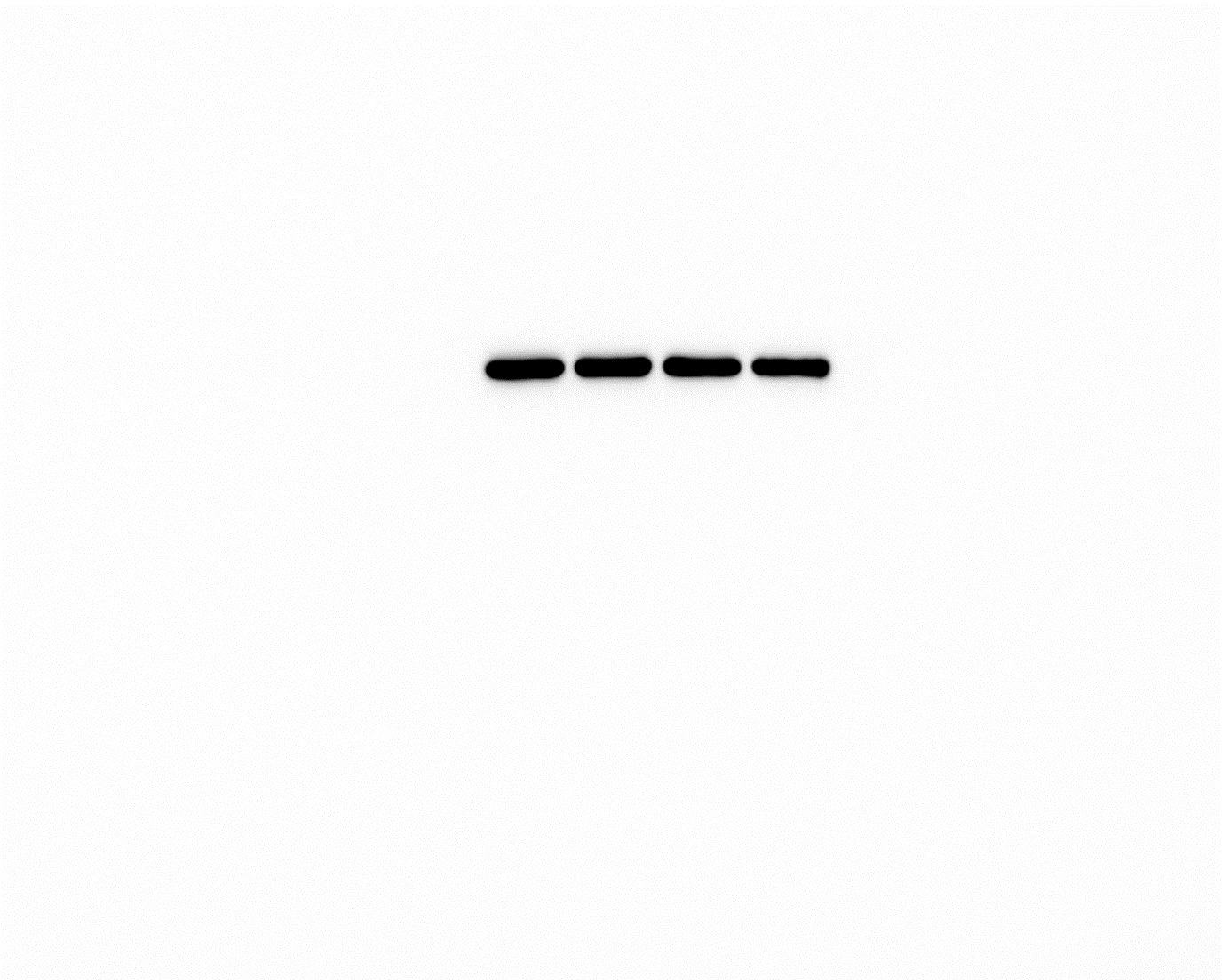

Supplement: Data S1 — The raw data of the MTT assays, the flow cytometry assays, Western Blot assays, and the animal studies. [file peerj-07-7760-s001.zip › raw data/Western Blot/GAPDH/GAPDH 2.tif]

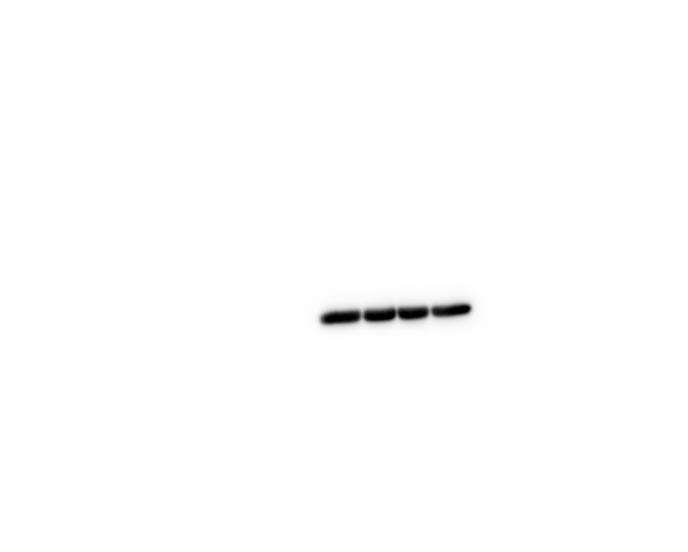

Supplement: Data S1 — The raw data of the MTT assays, the flow cytometry assays, Western Blot assays, and the animal studies. [file peerj-07-7760-s001.zip › raw data/Western Blot/GAPDH/GAPDH 3.tif]

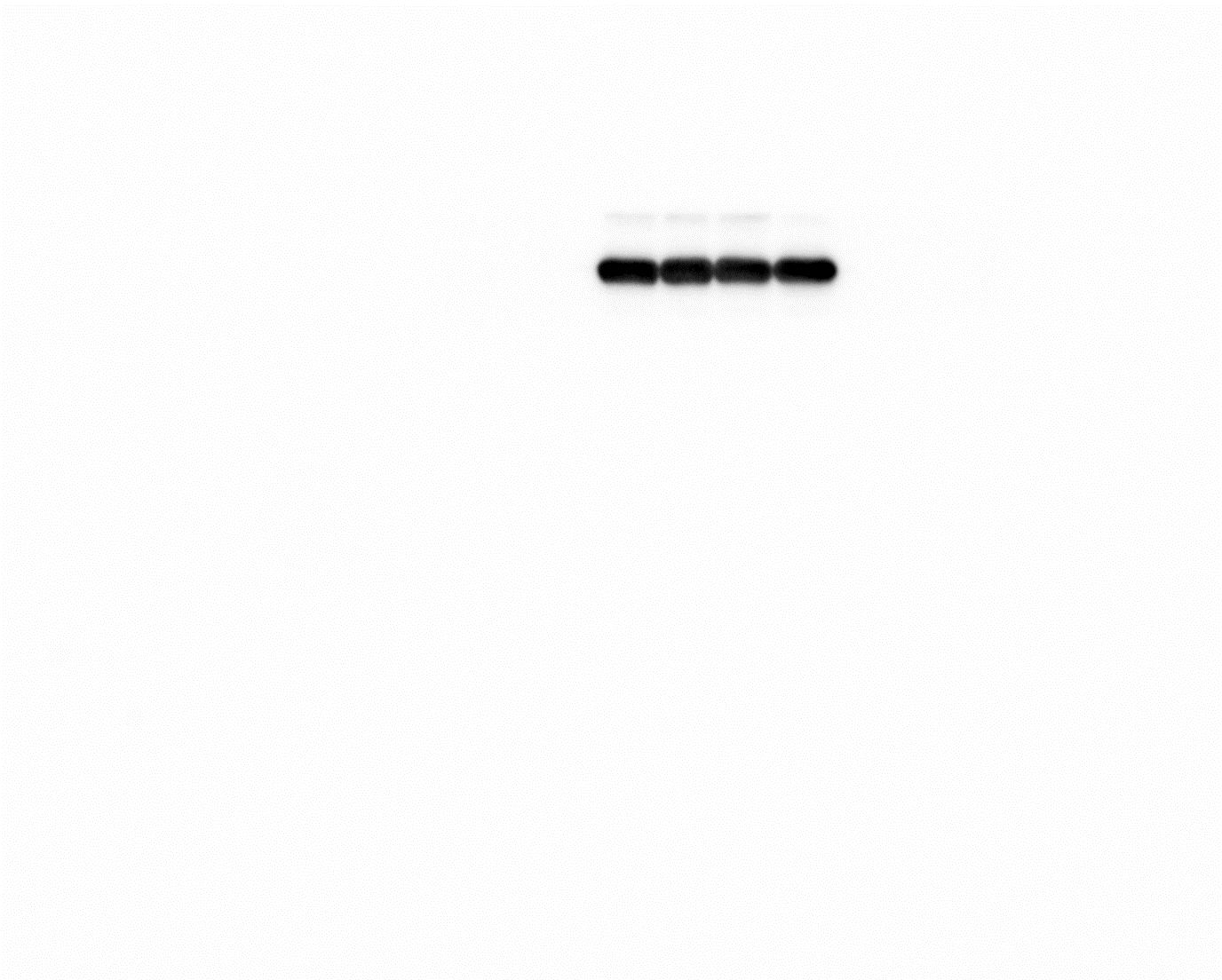

Supplement: Data S1 — The raw data of the MTT assays, the flow cytometry assays, Western Blot assays, and the animal studies. [file peerj-07-7760-s001.zip › raw data/Western Blot/GAPDH/GAPDH.tif]

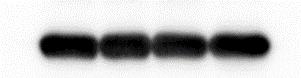

Supplement: Data S1 — The raw data of the MTT assays, the flow cytometry assays, Western Blot assays, and the animal studies. [file peerj-07-7760-s001.zip › raw data/Western Blot/GAPDH 1.jpg]

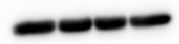

Supplement: Data S1 — The raw data of the MTT assays, the flow cytometry assays, Western Blot assays, and the animal studies. [file peerj-07-7760-s001.zip › raw data/Western Blot/GAPDH-2.JPG]

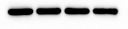

Supplement: Data S1 — The raw data of the MTT assays, the flow cytometry assays, Western Blot assays, and the animal studies. [file peerj-07-7760-s001.zip › raw data/Western Blot/GAPDH-3.JPG]

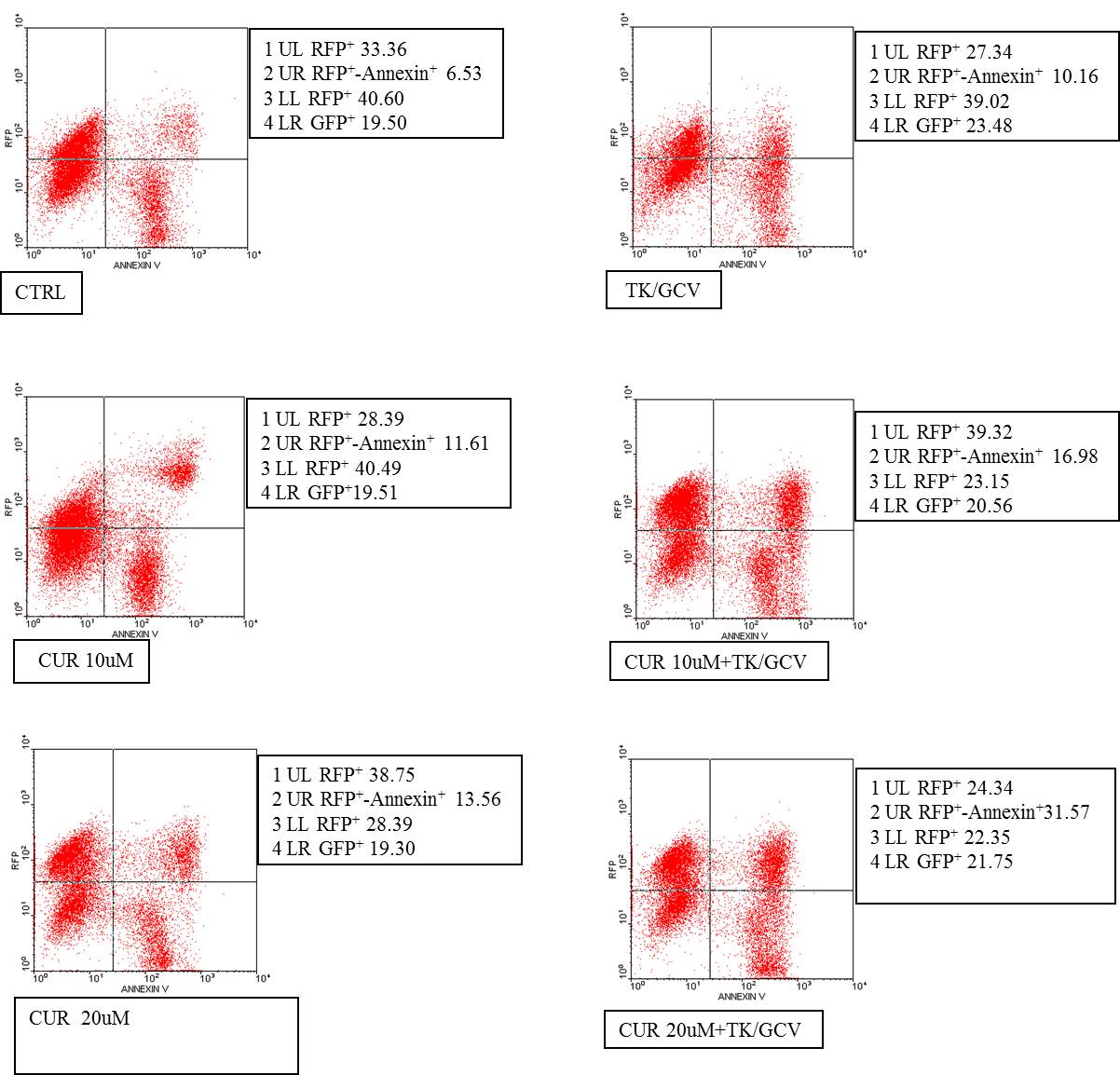

Supplement: Data S1 — The raw data of the MTT assays, the flow cytometry assays, Western Blot assays, and the animal studies. [file peerj-07-7760-s001.zip › raw data/bystander effect analysis/1.jpg]

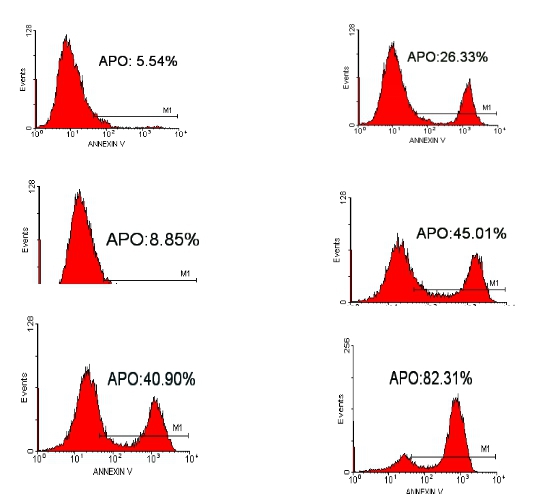

Supplement: Data S1 — The raw data of the MTT assays, the flow cytometry assays, Western Blot assays, and the animal studies. [file peerj-07-7760-s001.zip › raw data/bystander effect analysis/2.jpg]

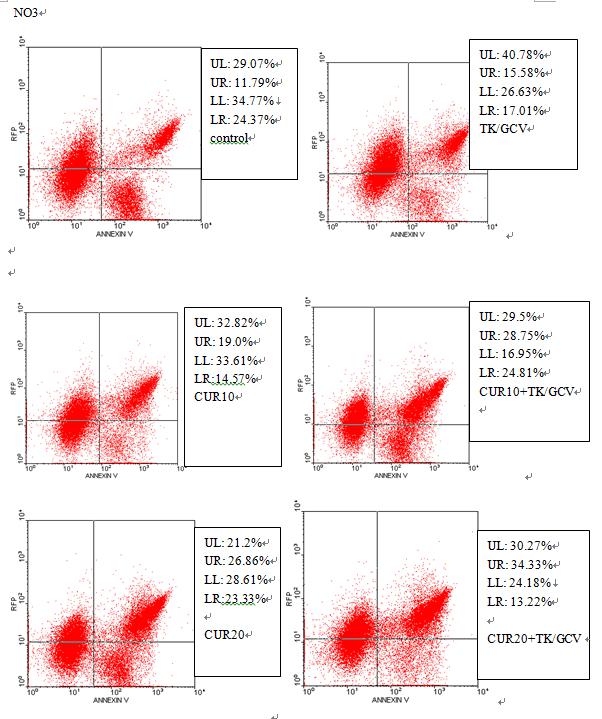

Supplement: Data S1 — The raw data of the MTT assays, the flow cytometry assays, Western Blot assays, and the animal studies. [file peerj-07-7760-s001.zip › raw data/bystander effect analysis/3.jpg]

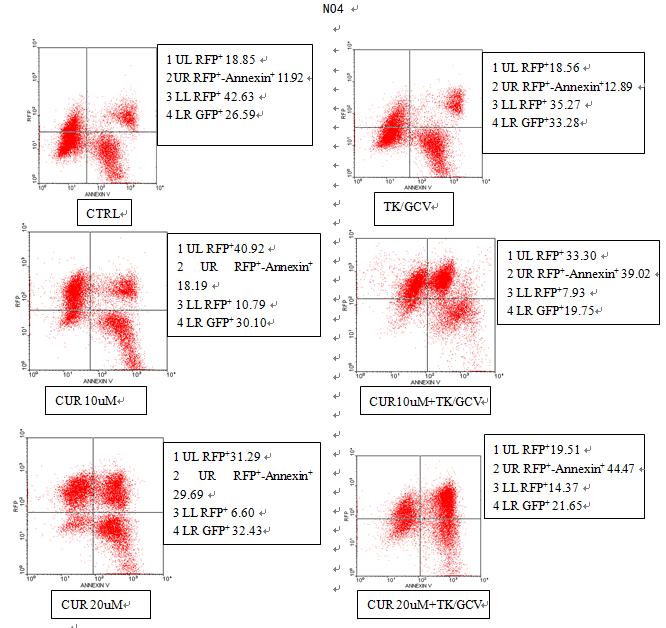

Supplement: Data S1 — The raw data of the MTT assays, the flow cytometry assays, Western Blot assays, and the animal studies. [file peerj-07-7760-s001.zip › raw data/bystander effect analysis/4.jpg]

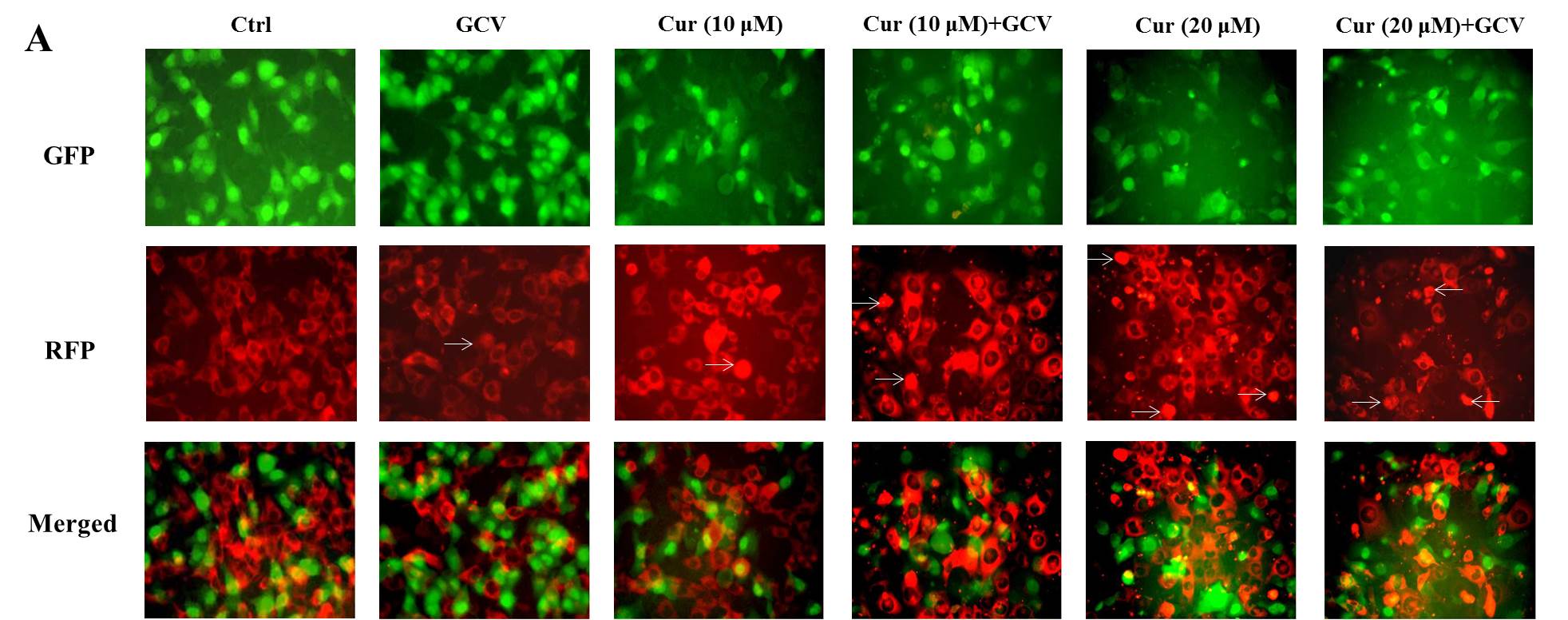

Supplement: Data S1 — The raw data of the MTT assays, the flow cytometry assays, Western Blot assays, and the animal studies. [file peerj-07-7760-s001.zip › raw data/bystander effect analysis/the red-green fluoresence images.jpg]

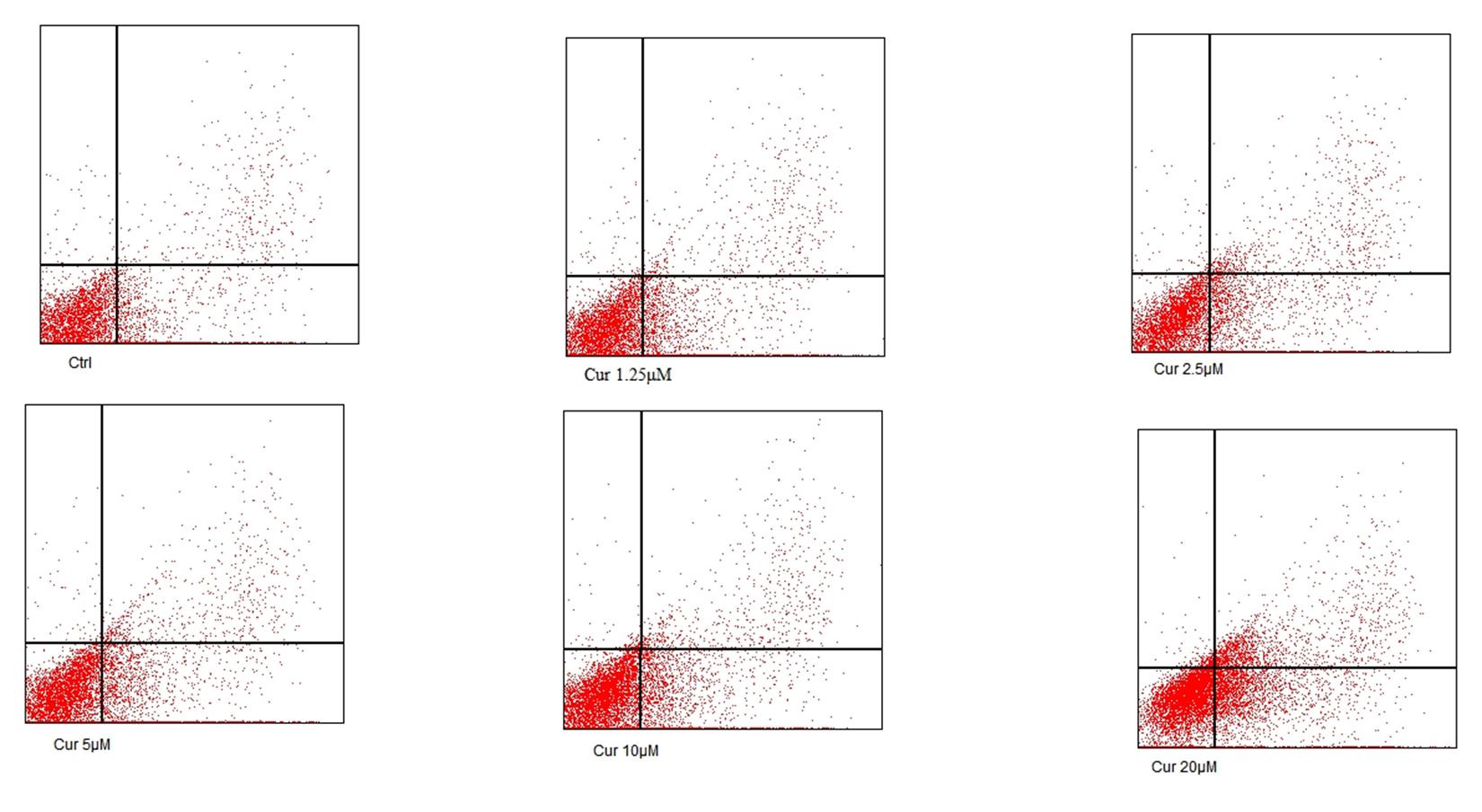

Supplement: Data S1 — The raw data of the MTT assays, the flow cytometry assays, Western Blot assays, and the animal studies. [file peerj-07-7760-s001.zip › raw data/dye transfer assay/dye transfer assay.jpg]

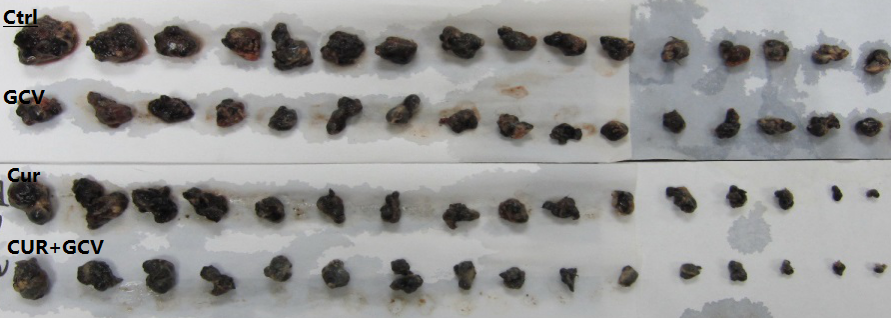

Supplement: Data S1 — The raw data of the MTT assays, the flow cytometry assays, Western Blot assays, and the animal studies. [file peerj-07-7760-s001.zip › raw data/in-vivo study/tumors image.png]
